# Catalytic liquefaction of human feces over Ni-Tm/TiO_2_ catalyst and the influence of operating conditions on products

**DOI:** 10.1016/j.enconman.2017.11.081

**Published:** 2018-02-01

**Authors:** Wenjia Wang, Lei Yang, Zhaosen Yin, Shengyan Kong, Wei Han, Jinglai Zhang

**Affiliations:** Renmin University of China, School of Environment and Natural Resources, Zhongguancun Avenue 59, Haidian District, Beijing, China

**Keywords:** Human feces, Hydrothermal liquefaction, Biocrude, Catalyst, Excrement treatment

## Abstract

In this study, human feces were hydrothermal liquefied and converted into biocrude over Ni-Tm/TiO_2_ catalyst. The influence of catalysts, reaction temperature, and holding time on the distribution of products and element content of biocrude was assessed. The biocrude yield increased to 53.16% with a reaction temperature of 330 °C, a holding time of 30 min, and adding Ni-Tm/TiO_2_ catalyst while the liquefaction conversion peaked at 89.61%. The biocrude had an HHV of 36.64 MJ/kg and was similar to heavy crude oil. The biocrude is rich in fatty acid amides, esters, and oxygen-containing-only heteroatom-ring compounds as well as some nitrogen-containing heteroatom-ring compounds. The main gaseous products were CO_2_, CH_4_, and C_2_H_6_. Hydrothermal liquefaction over Ni-Tm/TiO_2_ catalyst could be a potential method to handle human excrement treatment and produce biofuel.

## Highlights

• Catalytic liquefaction could convert 89.77% of human feces.
• The highest biocrude yield was 53.16% with Ni-Tm/TiO_2_ catalyst.
• Catalyst, temperautre and time significantly influenced the biocrude composition.
• The distribution of gaseous products at different HTL conditions were analyzed.

## 1 Introduction

An average adult human generates about 200 g of wet feces every day [32]. At present, 982 million of people still use open defecation while more 2.3 billion of people lack access to improved sanitation worldwide [39]. The human feces are rich in organic matters, parasite, and pathogenic bacteria. As the biochemical solid waste from human excretion, feces not only spread diseases burden but also show negative impacts on the environment by contaminating the water bodies, soils, and food sources [29]. Nevertheless, the human feces still contain various abundant chemical element to produce the valuable fertilizer and fuel. Therefore, the human excrement could be used as a renewable origin of the resource and energy [14], [22].

Traditional human feces utilization methods were universally applied through the history. For example, the composting has proved to be effective for killing both parasite and pathogenic bacteria while the anaerobic digestion is widely used for biogas production. However, these technologies require a long stabilization time to finish the biological treatment process, need additional treatment site, cause the unpleasant stink, and result in the derivative pollution from treatment. In comparison, the thermochemical process is a much faster and cleaner way to handle these human feces [12], [13], [27], [44]. Among various thermochemical methods, hydrothermal liquefaction (HTL) operates at conditions of a temperature of 200–350 °C and a pressure of 5–20 MPa, which is milder than that in pyrolysis and gasification [7], [17], [47]. Meanwhile, the HTL directly converts the wet biomass into biocrude without removing the water in biomass feedstock. Comparing with incineration and pyrolysis, the HTL method could save the operating cost from additional drying operation. Moreover, it should be noted that HTL process could kill pathogens at high temperature while producing the biocrude fuel. Therefore, great attention has been drawn to this research topic.

Previous studies showed that swine manure and human feces could be converted into biocrude, with a biocrude yield around 20–40% [9], [18], [40], [41]. However, the nitrogen (N), sulfur (S) and oxygen (O) content in biocrude are much higher than that in petroleum. The higher heteroatom (N, S, and O) content would cause catalyst poisoning or facility corrosion in biocrude refining for high-quality aircraft fuel. Thus, appropriate catalysts should be introduced into the HTL process to increase the biocrude yield or improve the biofuel properties. Various catalysts, such as alkalis, acids, transition metal oxides and noble metals were introduced into the HTL of other kinds of biomass. These catalysts showed the influence on the liquefaction conversion, biocrude yield and biocrude quality [16], [21], [30], [43], [46]. To the best of our knowledge, little information is available regarding the HTL of human feces, especially for catalytic liquefaction. In recent research, we found out that rare-earth-element and nickel-based TiO_2_ catalyst greatly improved the HTL of high-protein-content microalgae Spirulina [36]. This study convinced us that rare earth element and nickel supported on TiO_2_ catalyst should behave well in the application in HTL of human feces which contain plenty of protein. We hope this study could fill the research gap in catalytic HTL of human feces.

Catalytic HTL of human feces could reduce the pollution from the excrement to the environment and provide an added value for the outlet of human feces treatment. In this study, we would focus the catalytic liquefaction of human feces over Ni-Tm/TiO_2_ for producing biocrude and treating human feces. We also determined the influence of reaction temperature and holding time on the yields of products.

## 2 Materials and methods

### 2.1 Feedstock and reagents

Fresh human feces were collected from an aqua privy located in Xingshou Village, Nanzhuang Town, Changping District of Beijing, China. The feedstock was well stirred and then sealed and stored in cold storage at −12 °C in a freezer. The samples were transferred into a refrigerator overnight at 4 °C and thawed at 30 °C for 12 h before use. The characteristics of the human feces were presented in Table 1. The Tm(NO_3_)_3_·6H_2_O, Ni(NO_3_)_2_·6H_2_O, TiO_2_ powder, and HNO_3_ were all of the pure analytical grades and purchased from Shanghai Aladdin Bio-Chem Technology Co., Ltd. (Shanghai, China). Nitrate neodymium, lanthanum, and Cerium of analytic grade pure were purchased from Sinopharm Chemical Reagent Co., Ltd (Shanghai, China). Deionized water was used in the experiment. All the reagent were used without further purification.

**Table 1:** Proximate and ultimate analysis of feedstock.

Parameters	Human feces		Sludge [35]	Nannochloropsis [34]
	This study	[18]		
Proximate analysis (%)				
TS (Total solid)	15.13 ± 1.93	19.6 ± 3.8	15.48	–
Ash^a^	9.28 ± 0.42	17.0 ± 1.3	23.01	6.8
Biochemical analysis (%)				
Protein	45.28 ± 1.94	–	37.84	66.5
Lipid	13.50 ± 1.10	–	8.01	23.2
Carbohydrate	31.94 ± 0.89	–	31.14	10.3
Organic element analysis (%)				
C	50.51 ± 1.06	42.4 ± 1.3	46.68	47.08
H	6.75 ± 0.31	6.9 ± 0.9	6.85	8.77
O^b^	35.76 ± 0.90	43.1 ± 3.1	37.60	34.54
N	6.05 ± 0.49	5.9 ± 1.0	8.05	8.07
S	0.53 ± 0.02	1.7 ± 0.5	0.81	1.54

a Based on dry biomass.

b Calculated by difference.

### 2.2 Preparation of bimetallic catalyst

The catalyst preparation was based on our previous research [36]. Wet impregnation method was applied to prepare the catalyst precursor. TiO_2_ and the amounts of Tm(NO_3_)_3_·6H_2_O and Ni(NO_3_)_2_·6H_2_O needed stoichiometrically for catalyst contain 10 wt% Ni and 10 wt% Tm. The TiO_2_ was acidized by 1.0 mol/L nitric acid for 4 h, washed with deionized water and then dried at 105 °C for 8 h. The nickel and thulium precursors were dissolved in water and mixture together, and deionized water was added to the solution to bring the total volume to 2.5 mL of liquid per gram of dry TiO_2_. The solution was slowly dispensed onto the TiO_2_ support with continuous stir for 12 h. The impregnation liquid was separated from well-impregnated catalyst precursor by decantation. The wet catalyst precursor was dried at 105 °C for 24 h and then was calcinated in a muffle furnace at 700 °C for 4 h. The obtained catalyst was labeled as Ni-Tm/TiO_2_. XRF analysis determined that the obtained Ni-Tm/TiO_2_ catalysts consist of with 5.28% of NiO, 4.47% of Tm_2_O_3_ and 90.25% of TiO_2_. The catalysts of Ni-Nd/TiO_2_, Ni-La/TiO_2_, and Ni-Ce/TiO_2_ were prepared with the same method.

### 2.3 HTL process and product separation

The HTL process was carried out in a stainless 316 steel batch reactor (GS-0.6, Weihai Chemical Machinery Co., Ltd, China) with a volume capacity of 600 mL and heated by an external electrical furnace. The reactor is designed to a maximum temperature of 400 °C and pressure of 30 MPa. Human feces processed by HTL was carried out based on 300 g with/without the catalyst (based on 10% of dry weight of feedstock). The feces and catalyst mixture was stirred with a magnetic stirring and sealed in the reactor. Pure nitrogen was pressurized into the reactor headspace to provide an oxygen-free condition. The reactor was heated to a pre-set temperature, which was defined as the reaction temperature. Operating parameters, including reaction temperature and holding time, were investigated in the range of 250–350 °C and 0–720 min, respectively.

After holding for some time, the reactor was cooled down to room temperature. The pressure in the reactor was released from the gas outlet tube, and the gases were collected by a gas bag and weighted by an Analytical Balance (ME204T/02, METTLER TOLEDO, Switzerland). The gas bag was pre- vacuumized and weighted. 400 mL of Dichloromethane (DCM) was used to wash the reactor and the stirring head. The collected mixtures (containing the aqueous product, biocrude, solid residue, and DCM) were separated into the liquid phase mixtures and solid residue by vacuum filtration. The solid residue was washed for at least three times with 50 mL of DCM to remove other products. The solid residues were dried in a drying oven at 105 °C for 24 h and then weighed. The liquid phase mixture was separated into the aqueous product and the DCM-soluble phase product in a separating funnel. The DCM-soluble phase product was evaporated under vacuum (60 °C, 0.01 MPa) to remove the DCM. The obtained black sticky liquid was defined as the biocrude and weighted.

### 2.4 Calculation and analytic methods

All the calculated results listed are the average values from experimental results performed at least three times and all by dry ash free (daf) weight. Eqs. (1), (2), (3), (4), (5) calculated the yield of biocrude, solid residue, gaseous products, aqueous products and the liquefaction conversion, respectively.

(1) $$\text{Biocrude yield}\;\;({\text{Y}}_{\text{B}}\text{,}\%)=\frac{{\text{M}}_{\text{B}}}{{\text{M}}_{\text{F}(\text{daf})}}\times 100$$

(2) $$\text{Gaseous product yield}\;\;({\text{Y}}_{\text{G}}\text{,}\%)=\frac{{\text{M}}_{\text{G}}}{{\text{M}}_{\text{F}(\text{daf})}}\times 100$$

(3) $$\text{Solid residue yield}\;\;({\text{Y}}_{\text{s}}\text{,}\%)=\frac{{\text{M}}_{\text{S}}-{\text{M}}_{\text{A}}-{\text{M}}_{\text{C}}}{{\text{M}}_{\text{F}(\text{daf})}}\times 100$$

(4) $$\text{Aqueous product yield}\;\;(\%)=100-{\text{Y}}_{\text{B}}-{\text{Y}}_{\text{G}}-{\text{Y}}_{\text{S}}$$

(5) $$\text{Liquefaction conversion}\;\;(\%)=100-{\text{Y}}_{\text{S}}$$

where MB, MF (daf), MG, MA, and MC were the weight of biocrude, human feces (based on the dry ash-free weight), gaseous products, ashes in feces and catalyst. The liquefaction conversion represented the percentage of the organic matters in feces liquefied into non-solid phase product and was set to evaluate the treatment level of human feces.

The HHV of human feces and biocrude were calculated according to the Gumz correlation (Eq. (6)) [6]:

(6) HHV(MJ/kg)=0.3403C+1.2432H+0.0628N+0.1909S-0.0984O

where C, H, O, N and S were the weight percentage of carbon, hydrogen, oxygen, nitrogen, and sulfur in the feedstock and biocrude, respectively. Organic element content of the product was detected with an elemental analyzer (CE-400, Exeter Analytical, Inc. USA). The oxygen content was calculated by difference.

The energy recovery (ER) was used for evaluating the energy efficiency and calculated by Eq. (7):

(7) $$\text{ER}\;\;(\%)=\frac{\text{Biocrude yield}\;\times {\text{HHV}}_{\text{biocrude}}}{{\text{HHV}}_{\text{human feces}}}$$

The organic composition of biocrude was analyzed with a gas chromatography-mass spectrometry (GC-MS, QP2010, Shimadzu Co., Tokyo, Japan). A Varian DB-5 column (30 m × 0.25 mm × 0.25 μm) was the GC column, and helium was the carrier gas. The ion source temperature, injection temperature, and interface temperature were 200, 250 and 320 °C, respectively. The mass spectrometer was operated in positive electron impact mode (EI) at 70 eV with a scan range of *m*/*z* from 20 to650. All chromatogram peaks in spectra were compared with the electron impact mass spectrum from NIST Database (NIST11). Samples were directly diluted with acetone and filtered through a 0.45 μm filter. The column temperature was set at 50 °C for 2 min, then ramped up at a rate of 10 °C/min to 120 °C and maintained for 1 min, afterward increased to 250 °C at the same heating rate and maintained for 20 min.

The gaseous product analysis was carried out with an Agilent Technologies model 7820A gas chromatograph (GC) equipped with a thermal conductivity detector (TCD). The GC column was a 15 ft × 1/8 in. i.d. stainless steel column packed with 60 × 80 mesh Carboxen 1000 (Sperlco). Argon was used as the carrier gas. Mole fractions of the gaseous products were calculated based on the calibration curves, which was determined by the analysis of gas standards with known compositions.

## 3 Results and discussion

### 3.1 Effect of different catalysts in the liquefaction process

**Fig. 1:**
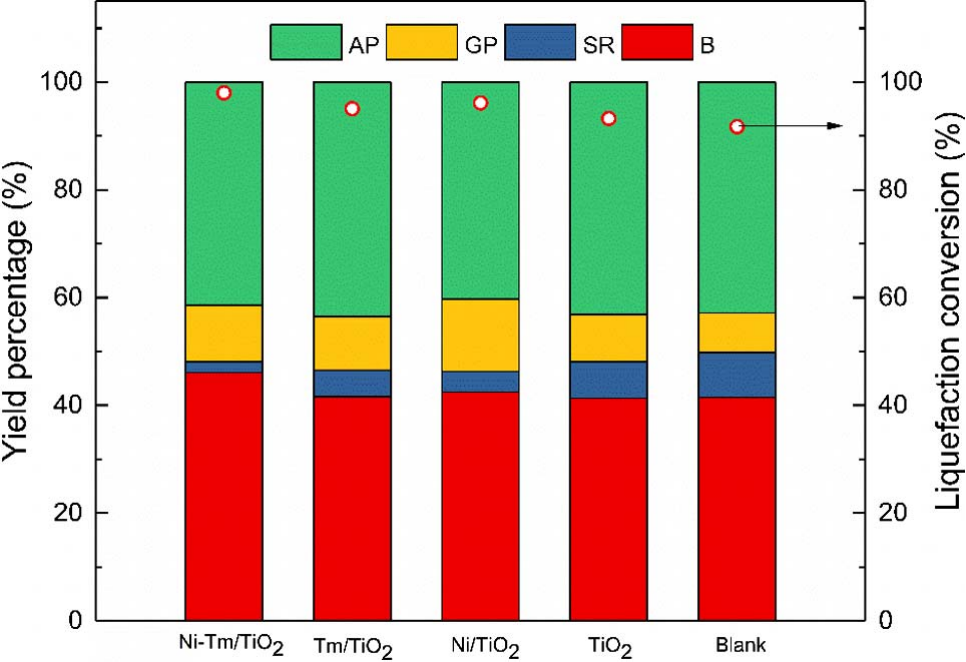
Products distribution and liquefaction conversion of human feces at different catalytic conditions. AP: aqueous product; GP: gaseous product; SR: solid residue; B: biocrude.

The introduction of catalysts into the HTL process could increase the biocrude yield and improve the biocrude quality. The preliminary experiments of feces HTL with different Ni-M/TiO_2_ catalyst (M = Tm, Nd, Ce, and La) suggested that only the Ni-Tm/TiO_2_ showed a positive effect on the HTL process (Fig. S1). Further discussion about the catalytic effect of the Nd, Ce, and La catalysts is beyond our research scope. Fig. 1 showed the effect of different catalyst conditions on the liquefaction process. The operating conditions were a reaction temperature of 300 °C, a holding time of 30 min, a biomass loading of 20% and with/without a catalyst loading of 10%. The process without catalyst was labeled as the blank experiment.

As shown in Fig. 1, the distribution of product yields was influenced by the introduction of catalysts. Adding Ni-Tm/TiO_2_ catalyst increased the biocrude yield from 41.57% to 46.09% while Ni/TiO_2_, Tm/TiO_2,_ and TiO_2_ demonstrated no significant effect on biocrude yields. Compared with the direct liquefaction of human feces in the literature, our biocrude yield was much higher, which could be due to the difference in feedstock composition [18]. According to further element analysis in Table 2, the Ni-Tm/TiO_2_ catalyst increased the concentration of carbon and hydrogen element by 10.39% and 4.64% in biocrude, respectively. The catalytic HTL process with Ni-Tm/TiO_2_ converted more than 64% of the carbon and 70% of the hydrogen in human feces into biocrude. Moreover, the H/C atom ratio of 1.54 indicated that the obtained biocrude was similar to heavy oil [20]. Meanwhile, the sulfur content in biocrude was reduced by 22.58% by adding Ni-Tm/TiO_2_ catalyst. This reduction was mainly attributed to the desulfuration of Ni catalyst, which was observed in the HTL of microalgae [8], [36]. The slight increase of oxygen content could come from the conversion of high-oxygen-content carbohydrate with the introduction of Tm and TiO_2_ catalyst composition. According to previous research, the carbohydrate needs higher active energy to be liquefied into biocrude and usually remained as the form of solid matters in the direct liquefaction process.[33]. However, the hydrocarbons like celluloses could be efficiently converted into biofuel over Tm catalyst and TiO_2_ catalyst [25], [37], [38]. That was also corresponding to the decrease of solid residue yield by 59.03% after the dosage of Ni-Tm/TiO_2_ catalyst. Adding Ni-Tm/TiO_2_ catalyst showed a positive influence on the improvement of liquefaction conversion, compared with the blank experiment.

**Table 2:** Elemental composition of biocrude samples from different catalysts. (300 °C, 30 min, 10 wt% catalyst loading).

Catalyst	Element composition (wt.%)					H/C	HHV (MJ/kg)	ER (%)
	C	H	O^a^	N	S			
Blank	71.10	9.79	11.31	7.49	0.31	1.65	35.78	66.77
TiO_2_	66.13	8.05	16.62	8.67	0.53	1.46	31.52	58.69
Ni/TiO_2_	68.39	8.76	14.44	7.96	0.45	1.54	33.33	63.50
Tm/TiO_2_	67.14	9.02	15.46	8.13	0.25	1.61	33.10	61.41
Ni-Tm/TiO_2_	71.79	9.24	10.45	8.28	0.24	1.54	35.45	73.34

ER: energy recovery.

Triplicate was conducted for element analysis, the relative standard deviation value was less than 1%, and only average value was presented.

a Calculated by difference.

The increased gaseous product yield indicated that Ni-Tm catalyst catalyzed more biomass into small gas molecules. The catalytic effect might be mainly attributed to the catalysis of nickel, which was the catalyst for the gas yield improvement in the HTL and the gasification of microalgae [3], [10], [23]. This improvement would be pleasing because some enhanced decarboxylation reactions can reduce the oxygen content in the form of CO_2_ with acceptable carbon loss. The CO_2_ was detected and in the gaseous product analysis in Section 3.4.

Therefore, Tm-Ni/TiO_2_ catalyst demonstrated a positive effect on hydrothermal liquefaction of human feces into biocrude by increasing both the biocrude yield and liquefaction conversion. Further discussion about optimizing the operating conditions of the application of the Ni-Tm/TiO_2_ catalyst was presented in detail in Section 3.2.

### 3.2 Effect of operating parameters on HTL process over Ni-Tm/TiO_2_ catalyst

#### 3.2.1 Effects of temperature on catalytic HTL of human feces over Ni-Tm/TiO_2_

**Fig. 2:**
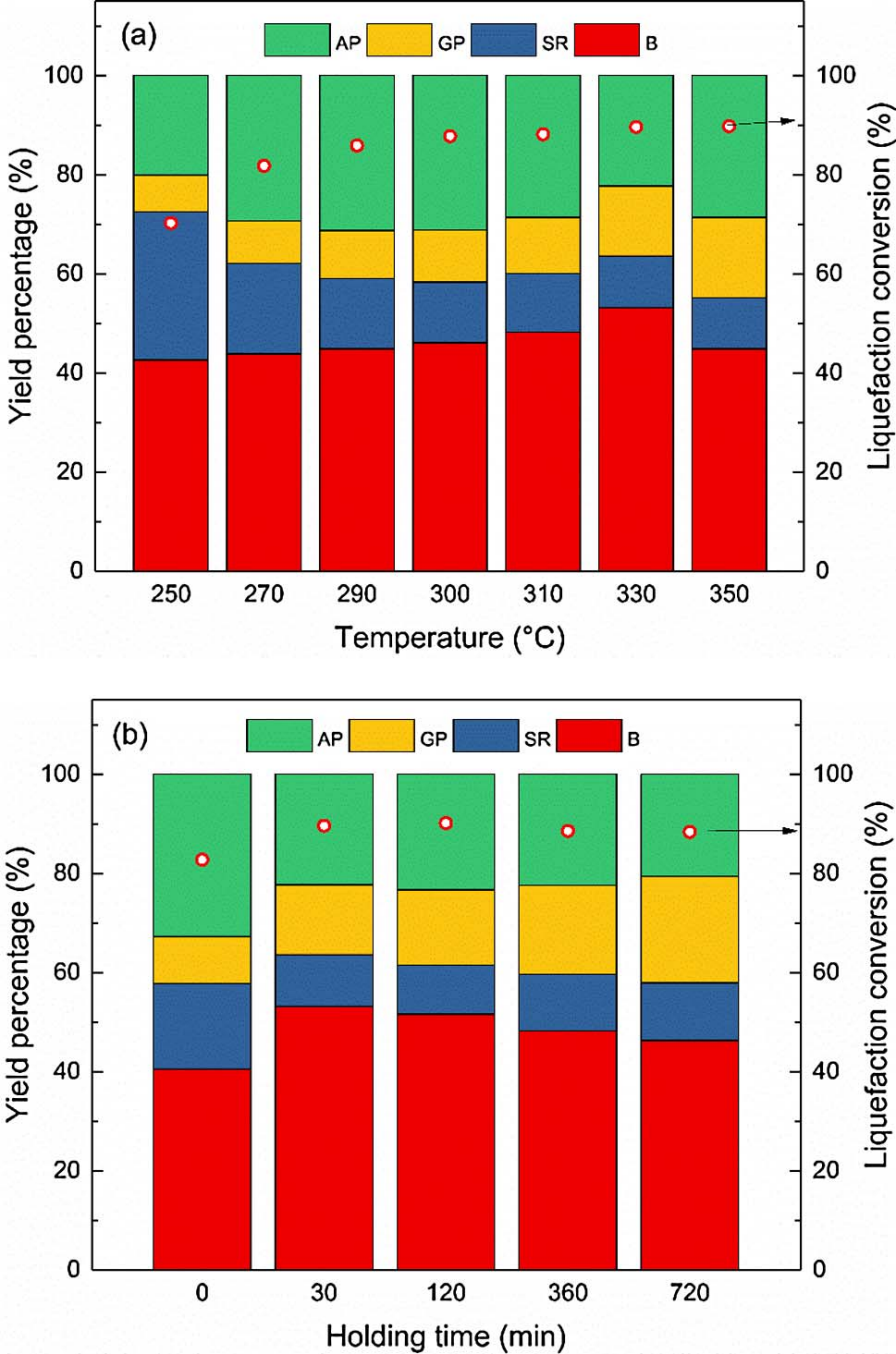
Products distribution and liquefaction conversion of human feces at various operating conditions (a) different reaction temperature (b) different holding time. AP: aqueous product; GP: gaseous product; SR: solid residue; B: biocrude.

The reaction temperature, as the most important operating parameter, showed a conclusive effect on the distribution of the product [1]. Fig. 2(a) presented the effect of temperature on HTL of human feces. All the HTL experiments were conducted at the operating conditions of a reaction time of 30 min, a Ni-Tm/TiO_2_ catalyst loading of 10 wt% and a reaction temperature range from 250 to 350 °C.

As shown in Fig. 2(a), with the reaction temperature increasing from 250 to 330 °C, the biocrude yield gradually walked to the highest peak (53.16%). Adding catalyst significantly improved the biocrude yield by 34.79%, compared with the biocrude yield of 39.44% without catalyst at 330 °C. The higher reaction temperature could provide the necessary energy to break the peptide bonds, C—C bonds, and C—O bonds in the biochemical compounds (carbohydrates, proteins, and lipids) into fragments and then enhance the formation of biocrude [21]. That could also explain the gradual decrease of the solid residue yield from 29.80 to 10.39% by the decomposition of solid organic matters. Interestingly, in this temperature range, the change of aqueous product yield showed a different trend. In contrast to the continuous increase or decrease in the yields of biocrude, gaseous products, and solid residues, the yield of aqueous products peaked from 20.13% to 31.30% at 290 °C and then gradually decreased to 22.29% at 330 °C. This trend could come from the changes of weights among the transformation of the different products along the general liquefaction reaction pathways investigated by Valdez [33]. When the reaction temperature increased from 250 to 290 °C, the yields of the biocrude and the gaseous products increased slightly, while the great decrease of yield of solid residues (from 29.80 to 15.27%) was close to the rapid growth of the yield of aqueous products (from 20.13 to 31.30%). When the reaction temperature was lower than 290 °C, the main transformation should be the decomposition of solid matters and the formation of aqueous products. However, in the temperature range from 290 to 330 °C, the formation of the biocrude and gaseous products from solid residues and aqueous products should be the protagonist among the liquefaction reaction pathways. Further increase of the temperature from 330 to 350 °C lowered the biocrude yield to 44.86%. Meanwhile, the yield of solid residues remained unchanged almost while both the yields of gaseous and aqueous products increased. The disappeared biocrude should be transferred into gaseous or aqueous products, because the higher reaction temperature could be in favor of the biomass gasification and might enhance the transformation from biocrude to aqueous products [4], [33]. Similar trends of product distributions under different temperatures were in accordance with previous works about the HTL of manure, microalgae, and macroalgae [2], [28], [40].

Further element analysis presented in Table 3 showed a steady increase of carbon and hydrogen contents with the temperature increasing. Raising reaction temperature could be conducive to the carbon and hydrogen, as the main element in the hydrocarbon fuel, transferring from feces to biocrude. The higher heat values (HHV) of biocrude samples suggested that raising reaction temperature led to a higher HHV and the highest HHV was obtained at 350 °C (36.76 MJ/kg). However, the higher reaction temperature could be a challenge to the equipment life and require a higher operating cost for more energy input. Taken the similar HHVs at 330 (36.64 MJ/kg) and 350 °C into the calculation, the appropriate reaction temperature should be 330 °C, which led to the highest energy recovery of 87.42%.

**Table 3:** Analysis of biocrude samples from various HTL operating conditions with Ni-Tm/TiO_2_ catalyst.

Temperature (°C)	Holding time (min)	Element content (wt.%)					H/C	HHV (MJ/kg)	ER (%)
		C	H	O^a^	N	S			
Blank									
300	30	71.10	9.79	11.31	7.49	0.31	1.65	35.78	66.76
330	30	72.83	9.84	10.60	6.44	0.29	1.62	36.43	64.48

With 10% catalyst									
250	30	68.69	8.69	13.45	8.64	0.53	1.52	33.50	64.08
270	30	70.13	8.73	13.35	7.34	0.45	1.49	33.95	66.72
290	30	70.58	8.30	13.81	6.95	0.36	1.41	33.48	67.37
300	30	71.79	9.24	10.45	8.28	0.24	1.54	35.45	73.34
310	30	71.56	9.74	11.66	6.82	0.22	1.63	35.78	77.42
330	30	72.86	9.83	9.43	7.42	0.46	1.62	36.64	87.42
350	30	72.89	9.89	9.23	7.48	0.51	1.63	36.76	74.01
330	0	64.00	8.67	17.03	9.44	0.86	1.63	31.64	57.48
330	30	72.86	9.83	9.43	7.42	0.46	1.62	36.64	87.42
330	120	73.48	10.02	8.94	7.13	0.43	1.64	37.11	85.88
330	360	73.47	10.13	8.15	7.92	0.33	1.65	37.35	80.86
330	720	73.68	10.37	8.43	7.05	0.47	1.69	37.67	78.19

ER: energy recovery.

Triplicate was conducted for element analysis, the relative standard deviation value was less than 1%, and only average value was presented.

a Calculated by difference.

#### 3.2.2 Effect of holding time on catalytic HTL of human feces over Ni-Tm/TiO_2_

Holding time is defined as a period for the reactor to react at a maximum temperature, excluding the heating and cooling times [42]. As an important operating parameter, the length of holding time is believed to be closely linked to the yield of biocrude and products of other phases [2]. Fig. 2(b) presented the effect of holding time on the HTL of human feces over Ni-Tm/TiO_2_ catalyst. The single factor experiments were carried out at 330 °C, a Ni-Tm/TiO_2_ catalyst loading of 10 wt% and with a holding time range from 0 to 720 min.

As shown in Fig. 2(b), the biocrude yield gradually increased from 43.48% to 53.16% when holding time extended from 0 to 30 min. It should be noted that the obtained biocrude yield with a holding time of 0 min much higher than that in other literature [11]. According to our experiment records, the heating of the reactor from 250 to 330 °C took around 30 min, which means that the experiment at 330 °C and 0 min could be regarded as an experiment holding for 30 min at a temperature between 250 and 330 °C. The solid residue yield of 17.03% at 0 min suggested the incomplete conversion of solid organic matter into products of other phases (liquid or gas) without enough reaction time. With further extension of holding time to 720 min, the biocrude yield gradually reduced to 46.25%. Meanwhile, the yield of solid residue and the aqueous product showed unchanged almost in this time range. However, the gaseous product yield was positively correlated with the length of holding time. The highest gas yield (21.47%) was obtained with a holding time of 720 min. It seems that holding for a long time gave the biomass and other products enough time to crack into small gaseous molecules [1]. Element analysis presented in Table 3 showed that a longer holding time resulted in a slight increase of element content of carbon and hydrogen, H/C atom ratio and HHV of biocrude in general. However, longer holding time (more than 30 min) led to a decrease in energy recovery from the highest at 30 min (87.42%) to 78.19% at 720 min. Moreover, the lengthened holding time also consumed more energy for heating. Therefore, a holding time of 30 min is appropriate for HTL of human feces over Ni-Tm/TiO_2_ catalyst.

### 3.3 Molecular characterization of biocrude from catalytic liquefaction

The GC-MS analyzed the biocrude samples obtained at 330 °C and 30 min, and the NIST library identified the main chemical compounds. It should be noted that some low-molecular-weight compounds could get lost during the evaporation process for obtaining the biocrude, while the high-molecular-weight components could not volatilize, then go through the GC column and be identified by GC-MS. Thus, we have admitted that GC-MS can characterize only part of components in biocrude, but the results could provide us some useful information about obtained biocrude. When identifying the biocrude, only compounds with identification probability more than 60% were selected and analyzed. The identification results were listed in Table 4. To simplify the discussion, the identified compounds were classified into six groups: esters, fatty acids, O-containing-only hetero-atom compounds (OH), amides, N-and-O-containing hetero-atom compounds (NOH), N-containing-only heterocyclic compounds (NH).

**Table 4:** Tentative identities and area percentage of major peaks in GC-MS for biocrude samples from HTL of human feces at 330 °C for 30 min and with and without 10 wt% Ni-Tm/TiO_2_.

Retention time (min)	Name	Molecular formula	Relative abundance (area %)	
			Blank	Ni-Tm/TiO_2_
3.49	Methylpyrazine	C_5_H_6_N_2_	0.33	2.07
4.38	2-Methyl-2-cyclopenten-1-one	C_6_H_8_O	1.87	–
4.41	2,3-Dimethylpyrazine	C_6_H_8_N_2_	0	1.95
5.39	2-Ethyl-5-methylpyrazine	C_7_H_10_N_2_	0.84	2.24
5.69	2-methyl-5-ethylpyridine	C_8_H_11_N	0	1.47
6.27	2,6-Diethylpyrazine	C_8_H_12_N_2_	0.54	–
7.10	2,3-Diethyl-5-methylpyrazine	C_9_H_14_N_2_	0.15	–
8.64	(E)-1-cycloheptenylpyrrolidine	C_11_H_19_N	0.17	–
12.12	2,2,2,2-(propane-1,3-diylbis(azanetriyl))tetraacetic acid	C_11_H_18_N_2_O_8_	–	3.03
13.04	7-ethylpentadecane-4,6-dione	C_17_H_32_O_2_	–	2.85
14.01	Methyl palmitate	C_17_H_34_O_2_	7.03	1.66
14.04	(3S,6S)-3,6-di-sec-butylpiperazine-2,5-dione	C_12_H_22_N_2_O_2_	–	2.39
14.4	Palmitic acid	C_16_H_32_O_2_	31.1	4.89
14.46	Phthalic acid, 3-methylbutyl undecyl ester	C_20_H_30_O_4_	6.35	1.12
14.71	Ethyl palmitate	C_18_H_36_O_2_	3.99	0.82
15.08	Phorbol	C_20_H_28_O_6_	1.45	1.41
16.86	(E)-octadec-1-enyl icosanoate	C_38_H_74_O_2_	–	1.76
17.02	Palmitamide	C_16_H_33_NO	4.85	12.66
18.05	1-Dodecanamide, N,N-dimethyl-	C_14_H_29_NO	5.51	–
19.48	(Z)-9-Octadecenamide	C_18_H_35_NO	2.82	10.63
22.08	Dioctyl phthalate	C_24_H_38_O_4_	1.64	8.42
29.68	Cholest-5-en-3-ol	C_27_H_46_O	–	19.22
Total relative abundance area			68.64	78.59

**Fig. 3:**
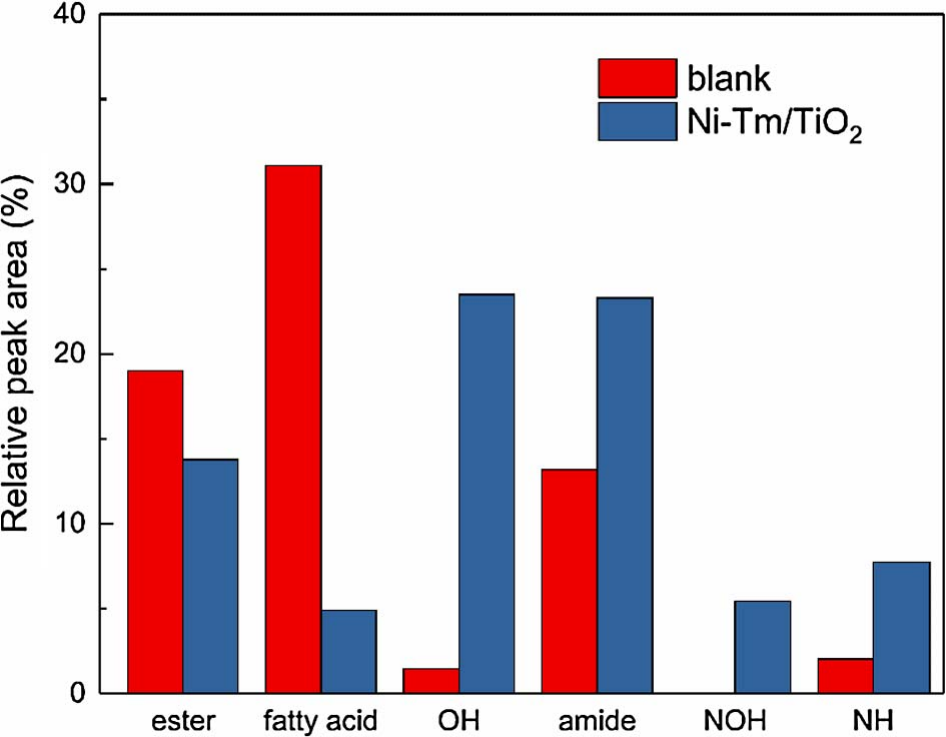
Classification of main compounds identified by GC-MS in the biocrude samples obtained at 330 °C and 30 min. OH: O-containing-only hetero-atom compound; NOH: N-and-O-containing hetero-atom compound; NH: N-containing-only heterocyclic compound.

As shown in Fig. 3, the biocrude produced without catalyst had the fatty acids as the uppermost compounds, followed by esters, fatty acid amides. There were a few NHs and OHs in this biocrude sample, and the identification showed the absence of the NOHs. By adding Ni-Tm/TiO_2_ catalyst, the relative content of esters and fatty acids decreased by 27.51% and 84.27%, respectively. Meanwhile, the relative content of fatty acid amides, which was the reaction product of fatty acid and small molecule amine, increased by 76.71%. The amines, as the decomposition product of amino acid, came from the hydrolysis of protein [5]. As both the products from fatty acids, there was an obvious competitive relationship between esters and fatty acid amides and the esterification reaction might be probably restrained by adding the Ni-Tm/TiO_2_ catalyst. According to Fig. 3 and Table 4, the relative content of NH and OH compounds in the catalytic biocrude sample increased from 2.03 and 1.45 to 7.73 and 23.48%, respectively. Besides, the NOH compounds appeared by adding the Ni-Tm/TiO_2_ catalyst. Some of these heterocyclic compounds could come from the Maillard reaction between the protein and carbohydrate [45]. This inference was corresponding to the catalytic effect on the carbohydrate conversion with Tm and TiO_2_ catalyst composition mentioned in Section 3.1. The identification results also indicated that the Ni-Tm/TiO_2_ catalyst must enhance the formation of compounds with more rings (like cholesterol) and the condensation to larger molecules (like phthalate, piperazinone, and tetraacetic acid) in the hot pressure water [15], [24], [26], [31]. The condensation reaction from smaller molecules to larger compounds could improve the biocrude yield, and lead to the deoxygenation, deamination, and desulfuration of biocrude. This conjecture was associated with the changed product distributions and element contents by adding Ni-Tm/TiO_2_ catalyst in Section 3.1.

### 3.4 Distribution of gaseous products

The identification and relevant fractions of gaseous products formed from HTL of human feces over Ni-Tm/TiO_2_ catalyst were discussed in this section. The most obtained compounds were N_2_ (initially loaded atmosphere gas), CO_2_, CO, CH_4_, C_2_H_6_, C_3_H_8_. Some unsaturated products like C_2_H_4_ and C_3_H_6_ were detected without catalyst application, and these ethylene and propene were likely hydrogenated to C_2_H_6_ and C_3_H_8_ during catalytic HTL. No H_2_, NH_3_, or NO_2_ was found out in all HTL experiments. The gas compositions were calculated on an N_2_-free basis.

**Fig. 4:**
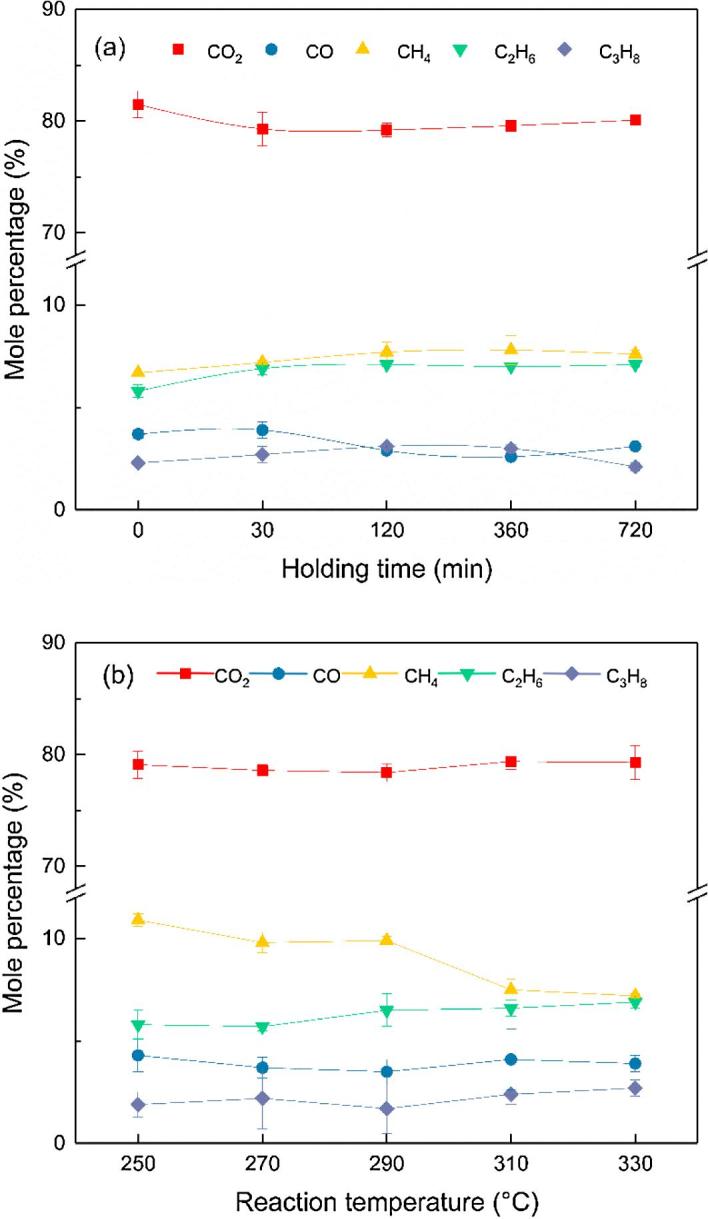
Gas composition from HTL of human feces over Ni-Tm/TiO_2_ catalyst: (a) 330 °C with different holding time; and (b) 30 min with different reaction temperature.

As shown in the Fig. 4, the distribution of gaseous product was affected by the extension of holding time and the increase of reaction temperature. CO_2_ was always with the highest amount under all conditions, as what demonstrated in the HTL of microalgae and soy protein concentration [8], [19]. The content of methane and ethane were the second and the third highest in relevant content. Fig. 4(a) shows the effect of holding time on the gaseous product distribution from HTL of human feces at 330 °C. The relevant content of methane and ethane increased while there was less CO_2_ with longer holding time until the holding time was longer than 120 min. Further extension of holding time lead to an almost unchanged relevant mole percentage of gases. Therefore, the gaseous product composition showed a weak influence from reaction time. Meanwhile, Fig. 4(b) suggested that the reaction temperature had a more strong influence on the gas composition. With the increase of temperature, the proportion of CH_4_ decreased, and the HTL process produced more C_2_H_6_. Moreover, the percentage of CO_2_ and C_3_H_8_ also increased slightly. This trend was similar to the gasification of biomass into small molecular alkane at a higher temperature with the nickel catalyst and various and complex organic reactions, like steam reforming, water-gas shift, methanation, and decarboxylation, were taken place during the HTL process [8], [10].

## 4 Conclusion

Human feces were treated by hydrothermal liquefaction over Ni-Tm/TiO_2_ catalyst. The best liquefaction conditions were at 330 °C and holding for 30 min, and the highest biocrude yield and liquefaction conversion were 53.16% and 89.61%, respectively. The Ni-Tm/TiO_2_ catalyst improved the biocrude yield by 34.79% and provided an energy recovery of 87.42%. Fatty acid amides, oxygen-containing-only heteroatom compounds, and esters were the main compounds in biocrude. CO_2_, CH_4,_ and C_2_H_6_ were the main gaseous products from catalytic liquefaction process. Catalytic hydrothermal liquefaction over Ni-Tm/TiO_2_ could be a potential method for the treatment and conversion of human feces.
