# Supplementary material for: Catalytic liquefaction of human feces over Ni-Tm/TiO2 catalyst and the influence of operating conditions on products
Source: Energy Convers Manag. 2018 Feb 1;157:239–45. doi: 10.1016/j.enconman.2017.11.081 (PMC5806599; doi:10.1016/j.enconman.2017.11.081)
Supplement: Supplementary data 1 [file mmc1.docx]

# Supplement information

## Fig.S1. Effect of different Ni-M/TiO_2_ catalyst on the biocrude yield from HTL of human feces (M =Tm, ND, Ce, and La)





## Fig.S2. the temperature curvy in the HTL reactor during the heating process with a reaction temperature of 330 °C
